# MRI features predict microvascular invasion in intrahepatic cholangiocarcinoma

**DOI:** 10.1186/s40644-020-00318-x

**Published:** 2020-06-23

**Authors:** Xijuan Ma, Liheng Liu, Jun Fang, Shengxiang Rao, Lulu Lv, Mengsu Zeng, Yibing Shi, Chun Yang

**Affiliations:** 1 Department of Radiology, Zhongshan Hospital, Fudan University, Fenglin Road 180# , Xuhui District, Shanghai, 200032 P.R. China; 2 Shanghai Institute of Medical Imaging, Shanghai, 200032 P.R. China; 3 Department of Radiology, Xuzhou Central Hospital, Xuzhou Clinical School of Xuzhou Medical University, No.199 Jiefang South Road, Quanshan District, Xuzhou, Jiangsu 221009 P.R. China; 4 Department of Radiology, Affiliated Kunshan Hospital of Jiangsu University, Kunshan, Jiangsu 215300 P.R. China

**Keywords:** Magnetic resonance imaging, Intrahepatic cholangiocarcinoma, Microvascular invasion

## Abstract

**Background:**

The presence of microvascular invasion (MVI) in intrahepatic cholangiocarcinoma (ICC) is a significant adverse prognostic factor. This study sought to investigate the correlation between preoperative imaging parameters and MVI in ICC.

**Methods:**

A total of 108 patients with surgically resected single ICC tumors (34 MVI-positive and 74 MVI-negative lesions) who underwent MRI examination, including T1WI, T2WI, DWI, and dynamic enhancement imaging, were enrolled in this retrospective study. The following qualitative and quantitative characteristics were evaluated: tumor morphology, signal features on T1WI and T2WI, intrahepatic duct dilatation, hepatic capsule retraction, target sign on DWI, dynamic enhancement pattern, arterial phase enhancement pattern, dot−/band-like enhancement inside the tumor, visible vessel penetration inside the tumor (hepatic artery, portal vein, or hepatic vein), integrity of the enhancement edge of the arterial phase, peripheral hepatic enhancement, tumor size, maximum enhancement edge thickness, arterial edge enhancement ratio, and delayed phase enhancement ratio. Other clinicopathological features were also used to predict and evaluate MVI in ICC. Chi-square test, Fisher’s exact test, and independent t-test were used for univariate analysis to determine the relationships among the presence of MVI and these MR parameters. Logistic regression analysis was used to identify predictors of MVI among these MR parameters.

**Results:**

Among MRI characteristics, tumor morphology, intrahepatic duct dilatation, arterial phase enhancement pattern, visible hepatic artery penetration sign, maximum diameter of the tumor and the arterial phase edge enhancement ratio were correlated with MVI (*P* = 0.007, 0.003, 0.008, 0.000, 0.003, and 0.002, respectively). Furthermore, higher CA19–9 levels (≥37 U/ml) and pathological tumor grade III were also related to MVI (*P* = 0.014 and 0.004, respectively). However, multivariate logistic regression analysis demonstrated that none of the parameters were independent risk factors for the diagnosis of MVI in ICCs.

**Conclusion:**

For the preoperative prediction of MVI in ICC, six qualitative and quantitative data obtained on preoperative MRI, as well as one tumorigenic marker and the pathological tumor grade, were statistically significant. More research is needed to identify MR characteristics that can be used as independent risk factors.

## Introduction

Intrahepatic cholangiocarcinoma (ICC), the second most common primary malignancy of the liver, is a subtype of cholangiocarcinoma that originates from the epitheliocytes of bile ductules [1]. ICC accounts for 20–25% of all cholangiocarcinomas and 10–15% of all primary hepatic malignancies, and its incidence is rising worldwide [2–4]. Based on its macroscopic appearance, ICC is classified into mass-forming, periductal infiltrating, intraductal growing, and mixed-type, with mass-forming ICC accounting for 60% of all ICCs [5, 6]. ICC patients have a poor prognosis, and surgical resection continues to be the only modality shown to prolong survival [7–9].

Research has revealed that microvascular invasion (MVI), tumor size, tumor grade, multiple tumors and lymph node positivity are associated with postoperative survival in patients with ICC [10]. MVI was defined as the presence of tumor in a portal vein, hepatic vein, or a large capsular vessel of the surrounding hepatic tissue lined by endothelium that was visible only on microscopy. Tsukamoto et al. reported that MVI is an independent predictor of ICC cure after hepatic resection [11]. Currently, MVI can only be diagnosed after postoperative pathology and can therefore be used only as a reference factor for adjuvant chemotherapy after ICC surgery [11]. Hence, the ability to predict MVI preoperatively is of particular importance. Unfortunately, few studies have explored the preoperative prediction of MVI in patients with ICC [12, 13], and their research focuses on diffusion-weighted imaging (DWI) and quantitative data on whether ADC values can predict MVI in ICC. As a single-center study, the primary objective of this study was to retrospectively analyze the clinicopathologic features and preoperative magnetic resonance (MR) imaging findings of 108 ICC patients undergoing primary surgery for a single tumor with the hope of discovering new MR imaging indicators for the preoperative prediction of MVI in ICC. This study is focused on mass-forming ICCs.

## Materials and methods

This retrospective diagnostic study was approved by the institutional review board. The requirement for written informed consent was waived.

### Patients

**Fig. 1:**
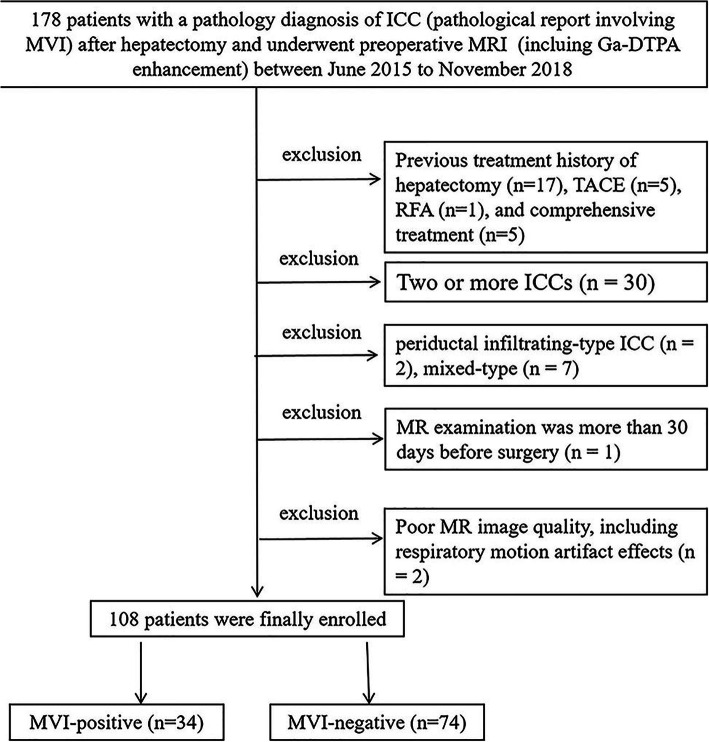
Flow diagram showing the inclusion and exclusion criteria for this study. ICC, intrahepatic cholangiocarcinoma; MVI, microvascular invasion; RFA, radiofrequency ablation; TACE, transcatheter arterial chemoembolization

During the period from June 2015 to November 2018, through a review of the hospital pathology and radiology database, a total of 178 patients (Fig. 1), all of whom were confirmed by postoperative pathology to have ICC (pathological report involving MVI) and to have undergone preoperative liver MR examination (including T1WI, T2WI, DWI, Gd-DTPA enhanced examination and excluding Gd-EOB-DTPA enhanced examination), were retrospectively identified. Considering that the chemical structures of Gd-EOB-DTPA and Gd-DTPA are different, the content of Gd in them is different, and the details of lesion enhancement such as the degree and thickness of ring-shaped enhancement in the arterial phase may be affected, so our study only selected patients examined with the same contrast agent (Gd-DTPA). Patients were enrolled if they met the following inclusion criteria: 1) no prior history of liver cancer treatment (including surgery and interventional therapy); 2) MR examinations performed within 30 days before surgery; 3) single mass; and 4) The MR image quality satisfied the diagnostic criteria (the image clearly showed the lesion, without obvious external artifacts, and respiratory motion artifacts did not affect the diagnosis), and the maximum lesion diameter was ≥0.5 cm. Of these 178 patients, 70 were excluded for the following reasons: previous treatment history (*n* = 28); two or more ICCs (*n* = 30); ICCs that were the periductal infiltrating-type (*n* = 2) or mixed-type (*n* = 7); the MR image was unavailable, acquired more than 30 days before surgery (*n* = 1) or of poor quality, including those with respiratory motion artifact effects (*n* = 2). In total, 108 patients with a single ICC were ultimately enrolled in this study (Fig. 1); these patients included 78 male and 30 female patients, with a sex ratio (M:F) of 2.6:1. Among the 108 patients, the imaging findings of 23 patients with ICC have been previously reported [12]. Compared with previous studies, which focused on the measurement of DWI and ADC quantitative values by one MR model, our research focuses on morphological data and a small amount of quantitative data from six different MR models in order to determine whether a correlation exists between MR imaging features and MVI.

### MR imaging

The 108 enrolled patients were scanned using the following different MR scanning systems: a 1.5-T UIHMR 560 scanner (Shanghai United Imaging Healthcare, China) was used for 42 patients; a 3.0-T UIHMR770 MR imager (Shanghai United Imaging Healthcare, China) was used for 27 patients; a Magnetom Avanto 1.5-T imager (Siemens Healthcare, Germany) was used for 21 patients; a Magnetom Aera 1.5 T imager (Siemens Healthcare, Germany) was used for 12 patients; a GE750 (3.0-T MR system, Discovery MR 750, GE Healthcare) was used for 4 patients; and a Magnetom Verio 3.0-T MRI System (Siemens Healthcare, Germany) was used for 2 patients. Taking UIHMR 560 scanner as an example, the scanning sequence and parameters are as follows. For conventional liver MRI, an anterior body phased-array coil and a posterior spine array coil were used for transverse (ie. axial) T2W breath-hold fat-suppressed fast spin echo sequence (T2W-FS) and transverse T1W breath-hold in-phase and opposed-phase gradient echo sequence (T1W IP-OP). DWI was performed using a breath-hold single-shot echo-planar imaging pulse sequence with b values of 0 and 500 mm^2^/s. For dynamic MRI, 0.2 mmol/kg Gd-DTPA was injected and immediately followed by a 20-ml saline flush through a power injector at a rate of 2 ml/s. Prior to the intravenous administration of contrast, a breath-hold fat-suppressed 3D T1W quick spoiled gradient echo sequence (T1W-FS) was performed to obtain the mask images.. Contrast-enhanced T1W-FS images, which included an transverse arterial phase, transverse portal venous phase, and coronal portal venous phase and transverse delayed phase, were obtained at 20–30 s (by monitoring, the scan is triggered when the contrast agent reaches the ascending aorta), 70–90 s, 100–120 s, and 160–180 s respectively after contrast medium injection. The detailed parameters used to obtain each acquisition sequence from the six scanners are shown in Table 1.

**Table 1:** MR imaging sequences and parameters from six scanners

Parameter	T2WI-FS	T1W IP-OP	T1W-FS tra	T1W-FS cor	DWI
**1.5-T UIHMR 560 scanner**					
Repetition time (msec)	2693	115.8	4.4–4.5	4.4–4.5	2807
Echo time (msec)	85.58	4.4 and 2.2	2.1–2.2	2.1–2.2	75.7
Matrix size	201 × 288	230 × 288	192 × 256	125 × 256	115 × 128
Field of view (mm2)	380 × 360	380 × 290	400 × 280	450X350	380 × 300
Slice thickness (mm)	6	6	3.5	3	6
Slice gap (mm)	1.2	1.2	0	0	1.2
Average	1	1	1	1	4
**3.0-T UIHMR770 MR**					
Repetition time (msec)	2000	4.27	3.3	3.3	4165
Echo time (msec)	106.2	2.5 and 1.21	1.5	1.5	66.3
Matrix size	256 × 256	288 × 168	320 × 216	288 × 208	128 × 101
Field of view (mm^2^)	346 × 346	300 × 300	270 × 270	340 × 340	300 × 300
Slice thickness (mm)	6–7	3–4	3	3–4	6–7
Slice gap (mm)	1.8–2.1	0	0	0	1.8–2.1
Average	1	1	1	1	5
**Avanto 1.5-T Siemens MR**					
Repetition time (msec)	3000–3500	112	5.04	5.13	2400–2600
Echo time (msec)	70–84	5.04 and 2.05	2.31	2.36	66
Matrix size	256 × 173	256 × 134	256 × 125	288 × 187	128 × 112
Field of view (mm^2^)	285 × 214– 308 × 380	285 × 214– 308 × 380	285 × 214– 308 × 380	350 × 350– 380 × 380	285 × 214– 308 × 380
Slice thickness (mm)	5–7	5–7	3–4	5	5–7
Slice gap (mm)	1–2.1	1–2.1	0	0	1–2.1
Average	1	1	1	1	1
**Aera 1.5 T Siemens MR**					
Repetition time (msec)	3500	230	4.38	4.36	3200
Echo time (msec)	84	2.38 and 4.76	1.93	2.03	56
Matrix size	320 × 224	320 × 240	320 × 240	320 × 320	128 × 128
Field of view (mm^2^)	380 × 308	380 × 278	380 × 297	350 × 350–380 × 380	380 × 308
Slice thickness (mm)	5.5	5.5	3–4	3	5.5
Slice gap (mm)	1.1	1.1	0	0	1.1
Average	1	1	1	1	1
**3.0-T GE750 MR**					
Repetition time (msec)	4500	3.60	3.54	3.95	5454
Echo time (msec)	88	2.23 and 1.12	1.67	1.84	49
Matrix size	320 × 224- 448 × 448	212 × 170- 256 × 192	212 × 170- 256 × 192	384 × 170	130 × 96
Field of view (mm^2^)	380 × 380–400 × 400	380 × 380–400 × 400	380 × 380–400 × 400	400 × 400–440 × 440	380 × 380–400 × 280
Slice thickness (mm)	6.5	5	5	4	6.5
Slice gap (mm)	1	1.1	0	0	1
Average	2	0.70	1.33	0.70	1
**Verio 3.0-T Siemens MRI**					
Repetition time (msec)	2000–3000	207	4.17	4.07	3400
Echo time (msec)	83	2.31 and 3.69	1.43	1.46	70
Matrix size	320 × 165	256 × 141	352 × 200	384 × 269	128 × 80
Field of view (mm^2^)	285 × 380–330 × 380	285 × 380–330 × 380	285 × 380–330 × 380	380 × 380	285 × 380–330 × 380
Slice thickness (mm)	5.5	5.5	3	3	6
Slice gap (mm)	1.1	1.1	0	0	1.8
Average	1	1	1	1	4

### Imaging analysis

All MR images were retrospectively analyzed together using a picture archiving and communication system (PACS; Pathspeed, GE Medical Systems Integrated Imaging Solutions, Prospect, IL) by two radiologists (XJ.M. and C.Y., who had 8 and 13 years of experience in abdominal imaging, respectively) who knew that the patient had ICC but did not know whether the lesion had MVI in pathological result. A third experienced abdominal radiologist with more than 20 years of experience was invited to resolve any disagreement between the two observers. Quantitative data were also measured by the two radiologists (XJ.M. and C.Y., respectively). The interobserver agreement was the agreement between the two observers, and the intraobserver agreement was the consistency of the results of the two readings by the senior radiologist (one-week interval).

### Qualitative analysis (morphological features of MR imaging)

**Fig. 2:**
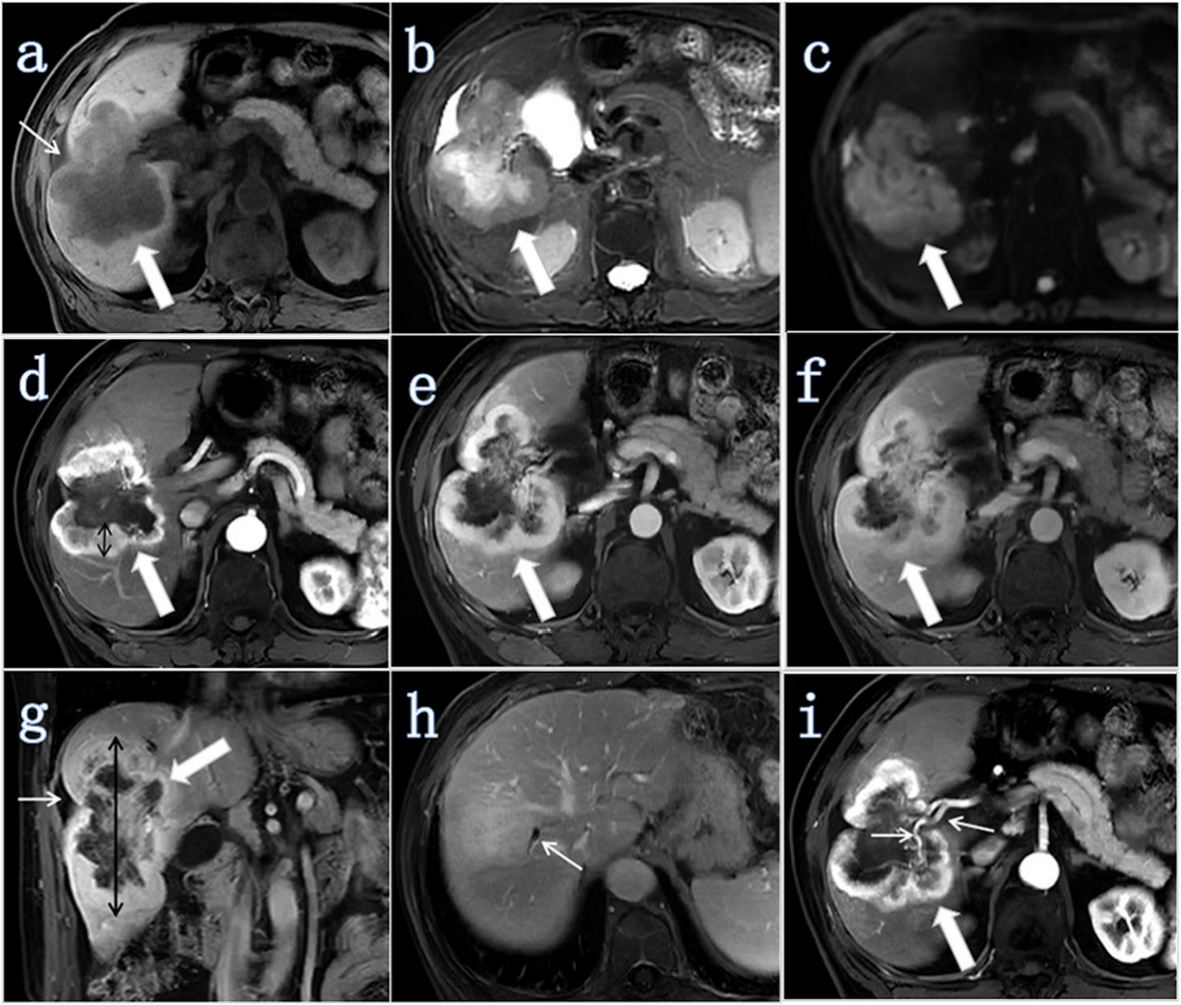
ICC (white thick arrow) with MVI in a 74-year-old man (tumor grade, G3; CA19–9, ≥37 U/ml). **a** Axial T1W-FS image showing tumor located in the right lobe of the liver and retraction of the hepatic capsule next to the tumor (white thin arrow). The tumor morphology was lobulated. **b** Axial T2W-FS image showing that the tumor had an unevenly high signal. The signal of the edge was slightly higher while the center signal was very high. **c** Axial DWI (b = 500 s/mm^2^) showing a higher signal without the target sign. **d** Axial arterial phase image showing that the tumor exhibited edged enhancement (ring high signal). The maximum enhancement edge thickness was 1.83 cm (black double arrow) and the arterial edge enhancement ratio was 15.56% (1.83/11.76). **e** Axial portal venous phase image. **f** Axial delayed phase image showing that the enhancement ratio of the lesion was approximately 2/4 (2/4 ≤ R < 3/4). **g** Coronal portal venous phase image showing retraction of the hepatic capsule next to the tumor (white thin arrow). The maximum diameter of the tumor was 11.76 cm (black double arrow). **h** Axial delayed phase image showing intrahepatic duct dilatation adjacent to the tumor (white thin arrow). **i** Axial arterial phase image showing visible hepatic artery penetration (white thin arrows)

**Fig. 3:**
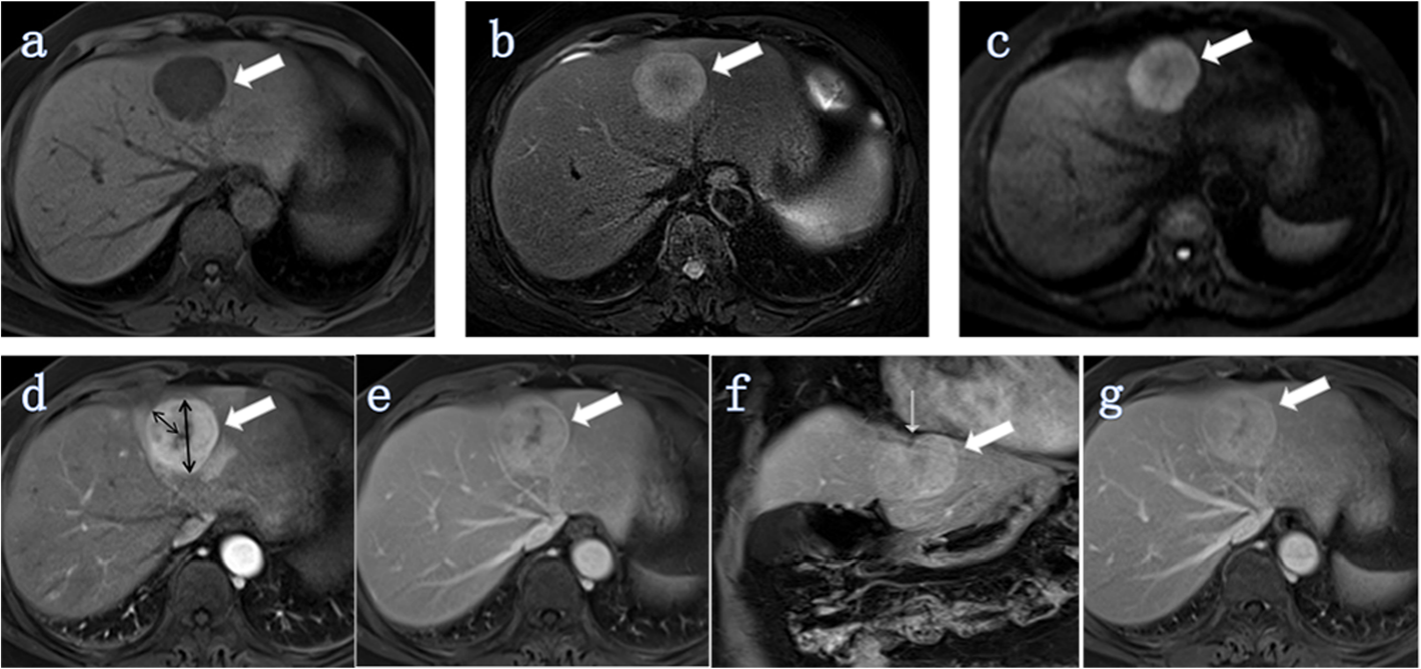
ICC (white thick arrow) without MVI in a 65-year-old woman (tumor grade, G2; CA19–9, ≥37 U/ml). Intrahepatic duct dilatation and visible hepatic artery penetration did not appear in all images of this lesion. **a** Axial T1-weighted image showing a tumor located in segment IV of the liver. The tumor morphology was spherical. **b** Axial T2-weighted-FS image showing that the signal of tumor edge was high intensity while that of the tumor center was low intensity. **c** Axial DW image (b = 500 s/mm^2^) showing a higher signal with a target sign. **d** Axial arterial phase image showing that the tumor exhibited edged enhancement (ring high signal). The maximum diameter of the tumor was 4.69 cm and the maximum enhancement edge thickness was 1.90 cm (black double arrows); the arterial edge enhancement ratio was 40.51% (1.90/4.69). **e** Axial portal vein phase showing the range of strengthening increased and began to fill the center of the lesion. **f** Coronal portal venous phase showing retraction of the hepatic capsule adjacent to the tumor (white thin arrow). **g** Axial delayed phase image showing that the signal of the tumor was high and the enhancement ratio of the lesion was approximately 4/4 (3/4 ≤ R ≤ 4/4)

**Fig. 4:**
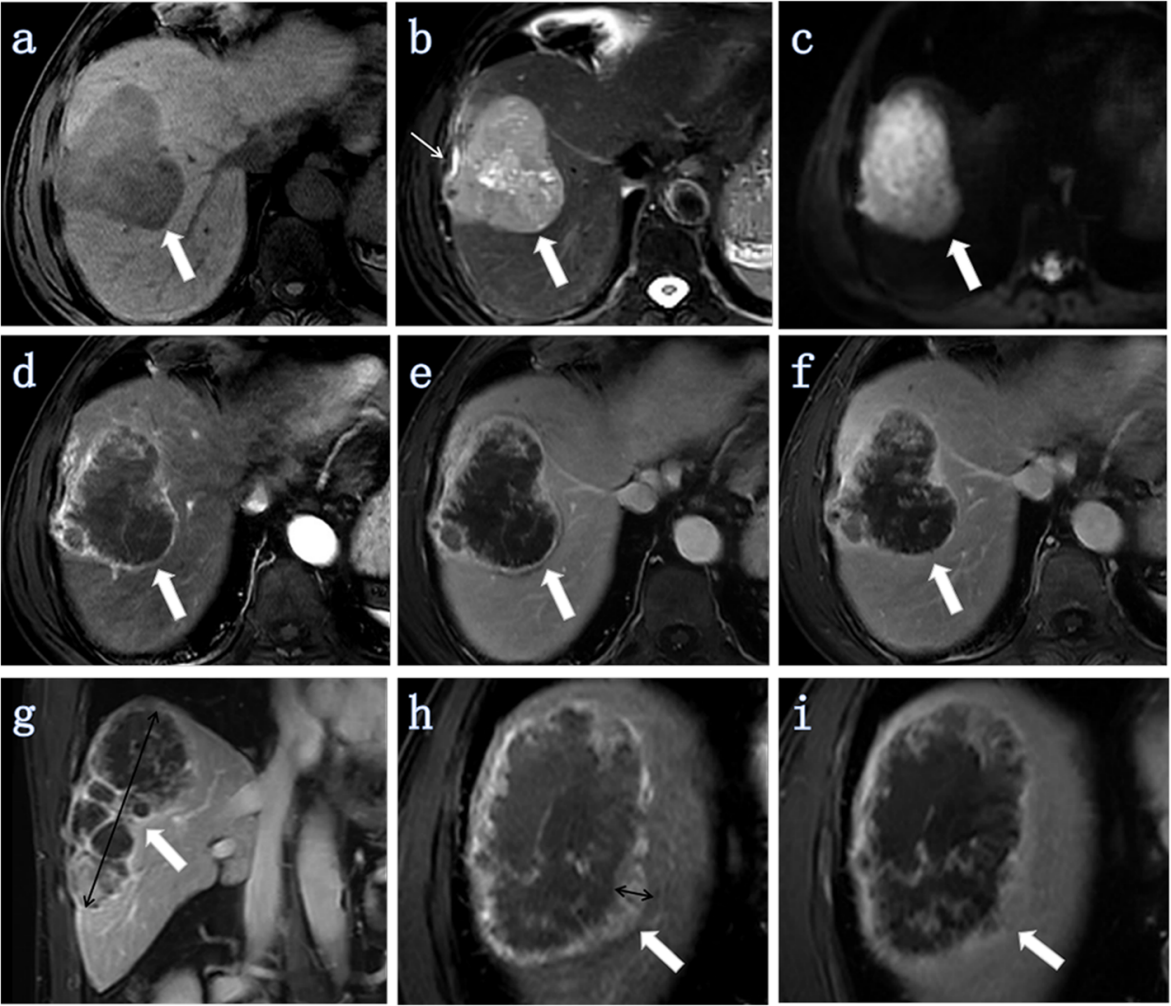
ICC (white thick arrow) with MVI in a 60-year-old man (tumor grade, G3; CA19–9, ≥37 U/ml (155.6 U/ml)). Intrahepatic duct dilatation and visible hepatic artery penetration did not appear in any images of this lesion. (a-f at the same level). **a** Axial T1W-FS image showing a tumor located in the right lobe of the liver. The tumor morphology was irregular. **b** Axial T2W-FS image showing the retraction of the hepatic capsule next to the tumor (white thin arrow) and the tumor was unevenly high signal. The signal of the edge was slightly higher while the center signal was very high. **c** Axial DWI (b = 500 s/mm^2^) showing an uneven higher signal without the target sign. **d** Axial arterial phase image showing that the tumor exhibited edged enhancement (ring high signal). **e** Axial portal venous phase image. **f** Axial delayed phase image showing that the enhancement ratio of the lesion was 0 ≤ R < 1/4. **g** Coronal portal venous phase image showing that the maximum diameter of the tumor was 12.13 cm (black double arrow). **h** Axial arterial phase image showing the maximum enhancement edge thickness was 0.34 cm (black double arrow), and the arterial edge enhancement ratio was 2.8% (0.34/12.13). **i** Axial delayed phase image on the same scanning level as figure **h**

**Fig. 5:**
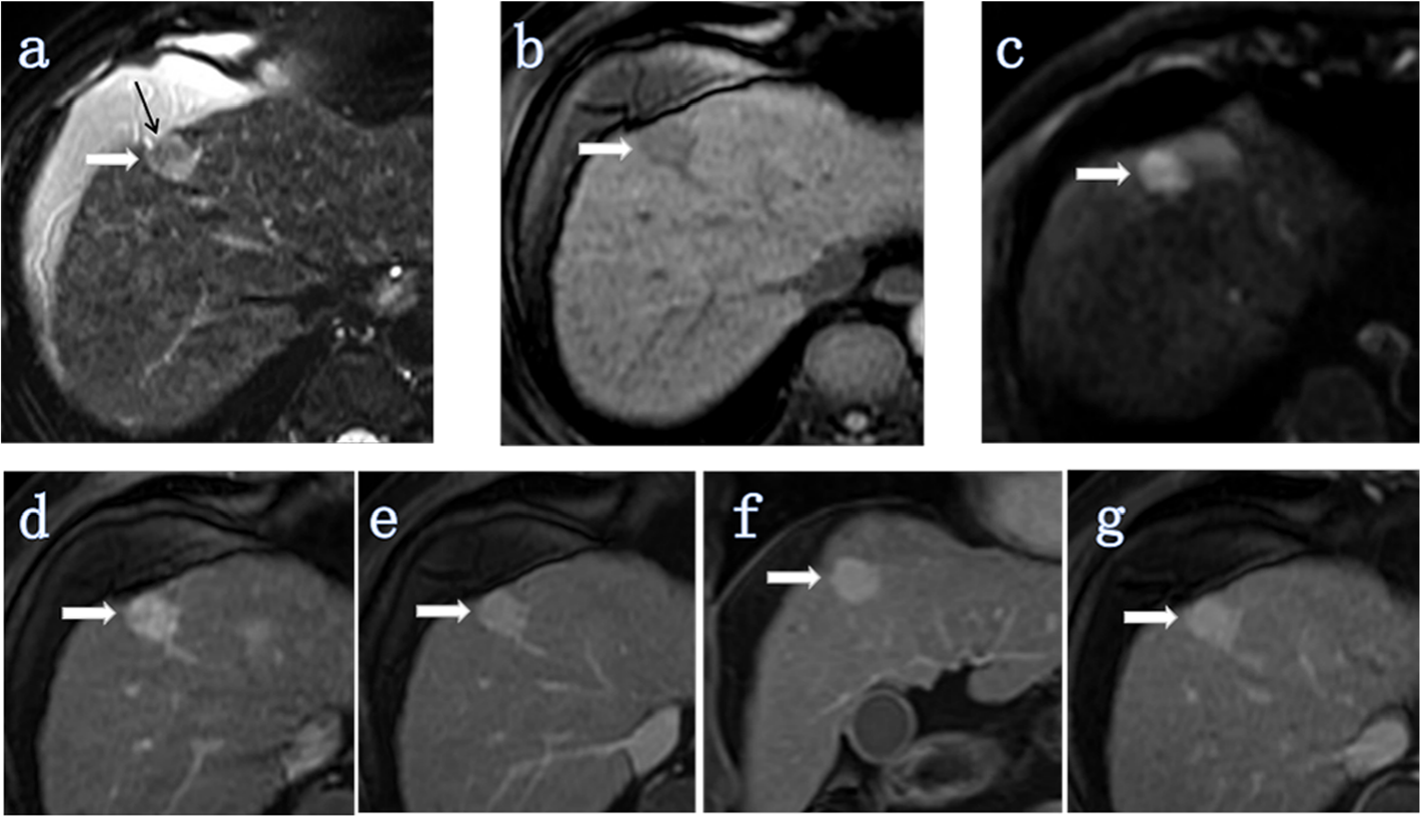
ICC (white thick arrow) without MVI in a 66-year-old man (tumor grade, G2; CA19–9, < 37 U/ml (20.6 U/ml)). Intrahepatic duct dilatation and visible hepatic artery penetration did not appear in all images of this lesion. **a** Axial T2-weighted image showing the signal of the tumor was unevenly high intensity and the retraction of the hepatic capsule adjacent to the tumor (black arrow). The tumor morphology was spherical. **b** Axial T1-weighted-FS image showing that the tumor was located in segment IV of the liver. **c** Axial DW image (b = 500 s/mm^2^) showing a uniform high signal. **d-g** Images during the arterial to delayed phase showing that the tumor showed continuous overall enhancement (high signal). The maximum diameter of the tumor was 1.89 cm and the maximum enhancement edge thickness was 1.89 cm, and the arterial edge enhancement ratio was 100% (1.89/1.89)

Qualitative analyses included the following (Figs. 2, 3, 4, 5): 1) tumor morphology (spherical/hemispherical/oval, lobulated/irregular); 2) relative signal intensity of the tumor compared to the surrounding liver parenchyma (hyperintense, isointense, and hypointense); 3) DWI (entirely uniform high signal, target sign); 4) intrahepatic duct dilatation within or outside of the lesion; 5) hepatic capsular retraction; 6) dynamic enhancement pattern (progressive enhancement, wash in-wash out, and other); 7) arterial phase enhancement pattern (it was classified as edge enhancement (ring enhancement), overall enhancement (overall high signal), partial enhancement (mixed signal), and no/mild enhancement (low signal)); 8) dot- or band-like enhancement inside the tumor [14] (in any phase of enhancement); 9) visible vessel penetration (hepatic artery, portal vein, or hepatic vein) inside the tumor (the presence of penetrating vessels in the lesion) [14]—We not only evaluated whether there were any visible blood vessels (hepatic artery, portal vein or hepatic vein) inside the tumor, but also evaluated whether each of these three blood vessels had a penetrating effect inside the tumor; 10) the integrity of the enhancement edge of the arterial phase (mainly with reference for the integrity of the enhanced edge of the edge-enhanced lesion; in addition, for the overall enhanced lesion, the enhancement edge was considered complete, while for no enhancement or mild enhancement or partially enhanced lesions, the enhanced edge was considered incomplete); and 11) peripheral hepatic enhancement (in any phase of enhancement): relatively high signal regions in the liver parenchyma adjacent to or surrounding the lesion, including wedge-, ring-, and irregular-shape.

### Quantitative analysis

Quantitative analyses were performed to evaluate tumor size, maximum enhancement edge thickness, the arterial edge enhancement ratio, and the delayed phase enhancement ratio (Figs. 2, 3, 4, 5).

Tumor size, which was defined as the maximum diameter, was measured on arterial phase transverse images or coronal portal venous phase images (The maximum diameter of irregular lesions may be displayed in the axial or coronal image: if the maximum diameter measured in the axial direction was greater than the maximum diameter measured in the coronal position, the axial measurement value was selected, and vice versa). Maximum enhancement edge thickness in the arterial phase was measured during imaging of the arterial phase: in edge-enhancing lesions, this value refers to the maximum thickness of the intensified edge; in lesions with overall enhancement, this value was considered to be the maximum diameter of the lesion; and in lesions with partial enhancement or no/mild enhancement, this value was considered to be zero. The arterial edge enhancement ratio (%, also referred to as the arterial ring enhancement ratio [14]) was defined as the ratio of the maximum enhancement edge thickness in the arterial phase to the maximum diameter.

The delayed phase enhancement ratio was defined as the ratio of the enhanced portion to the overall volume of the lesion during the delayed phase. Ratios were determined according to the visual method and were divided into four groups: 0 ≤ R < 1/4, 1/4 ≤ R < 2/4, 2/4 ≤ R < 3/4, and 3/4 ≤ R ≤ 4/4. All delayed phase enhancement ratios were determined by two observers. A third experienced abdominal radiologist was invited to resolve disagreements between the two observers.

### Clinical and pathological evaluation

Clinical data such as age, sex, history of hepatitis B, and tumor markers were collected from patient medical records. Recorded tumor markers included alpha-fetoprotein (AFP), carcinoembryonic antigen (CEA), and cancer antigen 19–9 (CA19–9).

The pathological information on 108 patients was acquired from pathology reports in our electronic medical records system. The MVI in ICC was evaluated on the basis of pathological reports and histological samples. The tumor grade recorded in the pathology report of each tumor and the liver background disease (including fatty liver, Edmondson-Steiner grade, and liver fibrosis and cirrhosis) were also recorded. MVI was defined as a tumor located within a vascular space lined by endothelium that was visible only by microscopy. The tumor pathological grade of ICC was evaluated using the following criteria: G1, well differentiated; G2, moderately differentiated; and G3, poorly differentiated [15]. When a single tumor contained regions exhibiting different degrees of differentiation, the “worst” grade was used as the index tumor grade.

### Statistical analysis

The maximum enhancement edge thickness values in the arterial phase were the mean values from the two observers. The agreement (inter- and intraobserver) of the thickness measurements was evaluated by calculating the intraclass correlation coefficient (< 0.21, poor; 0.21–0.4, fair; 0.41–0.6, moderate; 0.61–0.8, good; and 0.81–1, excellent). The above analyses were performed using the one-way random model intraclass correlation coefficient in the SPSS software package (v. 16.0; SPSS, Chicago, IL).

Other statistical analyses were performed using Stata 18.0 software (SPSS Inc., Chicago, IL, USA). Normally distributed data are expressed as the mean ± standard deviation, and comparisons between the two groups were performed using independent sample t tests. The skewed distribution data are expressed as the median (25, 75%), and comparisons between two groups were performed using rank sum tests. Comparisons between groups of categorical variables were performed by one-way analysis of variance. Parameters were analyzed using logistic regression to determine whether they were risk factors that predicted an MVI-positive diagnosis. A *P*-value less than 0.05 was considered to indicate a significant difference.

## Results

### Patient clinical and pathological characteristics

The comparisons of patient clinical and pathological characteristics according to MVI are shown in Table 2. The histopathological results revealed that 34 lesions were positive for MVI (mean age: 61.56 ± 9.97 years old, age range: 40–83 years old, sex ratio: M:F = 3.25:1), and 74 lesions were negative for MVI (mean age: 60.52 ± 11.53 years old, age range: 35–86 years old, sex ratio: M:F = 2.36:1). Significant differences were found in the level of the tumor marker CA19–9 (≥37 U/ml) and tumor grade (G2 and G3) between MVI-positive and MVI-negative patients (*P* = 0.014 and 0.004, respectively). The two groups were similar in their distributions of age, sex, HBV infection, AFP and CEA levels, Edmondson-Steiner grade, fibrosis stage, the presence of liver cirrhosis, and fatty liver (*P* > 0.05).

**Table 2:** Comparisons of patient clinical and pathological characteristics according to MVI

Variable/Parameter	MVI-positive (*n* = 34)	MVI-negative (*n* = 74)	*P* value
Mean age (years)^a^	61.56 ± 9.97	60.52 ± 11.53	0.6536
Age range (years)	40–83	35–86	
Sex ratio (M:F)	3.25:1	2.36:1	
HBV, n (%)			0.739
Active or convalescent stage	20 (58.82)	41 (55.41)	
Negative	14 (41.17)	33 (44.59)	
Tumor markers, n (%)			
AFP ≥ 20 ng/ml	4 (11.76)	8 (10.81)	1.000
AFP < 20 ng/ml	30 (88.24)	66 (89.19)	
CEA ≥ 5 ng/ml	11 (32.35)	16 (21.62)	0.232
CEA < 5 ng/ml	23 (67.65)	58 (78.38)	
CA19–9 ≥ 37 U/ml	21 (61.76)	27 (36.49)	0.014
CA19–9 < 37 U/ml	13 (38.24)	47 (63.51)	
Pathology findings of background liver and tumor, n (%)			
Edmondson-Steiner grade			0.886
G0	15 (44.12)	34 (45.95)	
G1-G2	17 (50.00)	37 (50.00)	
G3-G4	2 (5.88)	3 (4.05)	
Fibrosis stage			0.472
S0	14 (41.18)	36 (48.65)	
S1-S2	10 (29.41)	24 (32.43)	
S3-S4	10 (29.41)	14 (18.92)	
Liver cirrhosis (S4)	5 (14.71)	9 (12.16)	0.715
Noncirrhotic (S0-S3)	29 (85.29)	65 (87.84)	
Fatty liver, n (%)	5 (14.71)	12 (16.22)	0.819
No fatty liver, n (%)	29 (85.29)	61 (82.43)	
Tumor grade G2	4 (11.76)	29 (39.19)	0.004
Tumor grade G3	30 (88.24)	45 (60.81)	

The data are presented as the number (%) of patients

^a^Data are shown as the mean ± standard deviation

### MR findings

The agreement (inter- and intraobserver) of the maximum enhancement edge thickness values in the arterial phase were both significant (intraclass correlation coefficient: 0.997; 95% CI: 0.995, 0.998; intraclass correlation coefficient: 0.999; 95% CI: 0.999, 0.999). The measurements of the senior radiologist were selected as the data in the analysis. The radiological features of the ICCs are presented in detail in Table 3 and Table 4. Among the recorded MRI characteristics, tumor morphology (*P* = 0.007), intrahepatic duct dilatation (*P* = 0.003), arterial phase enhancement pattern (*P* = 0.008), visible hepatic artery penetration (*P* = 0.000), tumor maximum diameter (*P* = 0.003), and the arterial edge enhancement ratio (*P* = 0.0002) were significantly associated with MVI, while the other features analyzed were not.

**Table 3:** Comparison of qualitative data obtained on MR plain scan according to MVI in 108 ICCs

	MVI-positive (*n* = 34)	MVI-negative (*n* = 74)	*P* value
Tumor morphology			
Spherical/hemispherical/oval	6 (17.65)	33 (44.59)	0.007
Lobulated/irregular	28 (82.35)	41 (55.41)	
Signal on T1WI			0.589
Low intensity	32 (94.12)	72 (97.30)	
Isointensity/High intensity	2 (5.88)	2 (0.27)	
Signal in T2WI-FS			0.178
Low intensity/Isointensity	0 (0)	5 (6.76)	
High intensity	34 (100)	69 (93.24)	
Entire/uniform high intensity	20 (58.82)	27 (36.49)	0.059
Edge high intensity	14 (41.18)	42 (56.76)	
Signal in DWI (b = 500 s/mm^2^)			0.739
Entirely uniform high signal	20 (58.82)	41 (55.41)	
Target sign on DWI	14 (41.17)	33 (44.59)	
Intrahepatic duct dilatation	21 (61.76)	23 (31.08)	0.003
No biliary dilation	13 (38.24)	51 (68.92)	
Hepatic capsular retraction	17 (50)	27 (36.49)	0.184
No hepatic capsular retraction	17 (50)	47 (63.51)	

The data are presented as the number (%) of patients

**Table 4:** Comparison of qualitative and quantitative Gd-DTPA enhancement MR imaging features according to MVI

	MVI-positive (*n* = 34)	MVI-negative (*n* = 74)	*P* value
Dynamic enhancement pattern			0.870
Progressive	27 (79.41)	56 (75.68)	
Wash in-wash out	4 (11.76)	9 (12.16)	
Other	3 (8.82)	9 (12.16)	
Arterial phase enhancement pattern			0.008
Edged enhancement (ring high signal)	28 (82.35)	62 (83.78)	
Overall enhancement (high signal)	1 (2.94)	11 (14.86)	
Partial enhancement (mixed signal)	2 (5.88)	0 (0)	
No/mild enhancement (low signal)	3 (8.82)	1 (1.35)	
Dot−/band-like enhancement inside the tumor	26 (76.47)	44 (59.46)	0.086
Absent	8 (23.53)	30 (40.54)	
Visible vessel penetration	25 (73.53)	42 (56.76)	0.095
Absent	9 (26.47)	32 (43.24)	
Visible hepatic artery penetration	12 (35.29)	6 (8.11)	0.000
Absent	22 (64.71)	68 (91.89)	
Visible portal vein penetration	15 (44.12)	29 (39.19)	0.628
Absent	19 (55.88)	45 (60.81)	
Visible hepatic vein penetration	17 (50)	25 (33.78)	0.108
Absent	17 (50)	49 (66.22)	
Peripheral hepatic enhancement	18 (52.94)	43 (58.11)	0.615
Absent	16 (47.06)	31 (41.89)	
Maximum diameter (cm)^a^	6.38 ± 3.06	4.45 ± 2.13	0.0003
Maximum diameter ≤ 5 cm^b^	13 (38.24)	48 (64.86)	0.010
Maximum diameter > 5 cm	21 (61.76)	26 (35.14)	
Maximum enhancement edge-thickness in arterial phase (mm)^b^	3.95 (2.5, 14.3)	8.9 (3.8, 17.1)	0.0538
Arterial enhanced edge integrity	16 (47.06)	47 (63.51)	0.107
Arterial enhanced edge is incomplete	18 (52.94)	27 (36.49)	
Arterial edge enhancement ratio (%)^b^	9.47 (4.26, 27.22)	21.48 (11.46, 42.19)	0.0002
Delayed phase enhancement ratio			1.000
0 ≤ R < 1/4	5 (14.71)	10 (13.51)	
1/4 ≤ R < 2/4	1 (2.94)	2 (2.70)	
2/4 ≤ R < 3/4	5 (14.71)	10 (13.51)	
3/4 ≤ R ≤ 4/4	23 (67.65)	52 (70.27)	

The data are presented as the number (%) of patients

^a^Data are shown as the means±standard deviation. ^b^Data are shown as the median (25% percentile, 75% percentile)

### Univariate and multivariate analyses

Univariate logistic regression analysis revealed eight risk factors that were significantly related to the MVI of ICCs (Table 5): CA19–9 ≥ 37 U/ml, tumor grade (G3), tumor morphology (lobulated/irregular), intrahepatic duct dilatation, arterial phase enhancement pattern, visible hepatic artery penetration, maximum diameter, and arterial edge enhancement ratio (*P* = 0.016, 0.007, 0.009, 0.003, 0.011, 0.001, 0.001, and 0.010, respectively). The other parameters analyzed were not significantly correlated with MVI.

**Table 5:** Univariate and multivariate analyses of risk factors for MVI of ICCs

Risk Factor	Univariate Analysis		Multivariate Analysis	
	Odds Ratio (95% CI)	*P* value	Odds Ratio (95% CI)	*P* value
Age (years)	1.047 (0.980–1.118)	0.650	…	…
HBV	1.100 (0.201–6.023)	0.739	…	…
AFP ≥ 20 ng/ml	0.166 (0.012–2.342)	0.884	…	…
CEA ≥ 5 ng/ml	0.832 (0.148–4.684)	0.234	…	…
CA19–9 ≥ 37 U/ml	1.991 (0.349–11.355)	0.016	1.542 (0.552–4.310)	0.409
Edmondson-Steiner grade	0.068 (.004–1.114)	0.668	…	…
Fibrosis stage	25.104 (1.953–322.686)	0.275	…	…
Liver cirrhosis	0.207 (0.0140–3.065)	0.715	…	…
Fatty liver	0.961 (0.158–5.851)	0.841	…	…
Tumor grade	10.942 (1.664–71.956)	0.007	3.076 (0.900–10.515)	0.073
Tumor morphology	1.493 (0.277–8.038)	0.009	1.316 (0.383–4.529)	0.663
Intrahepatic duct dilatation	1.463 (0.335–6.387)	0.003	1.497 (0.531–4.215)	0.445
Hepatic capsular retraction	1.100 (0.258–4.685)	0.186	…	…
Target sign on DWI	0.812 (0.191–3.446)	0.739	…	…
Dynamic enhancement pattern	4.020 (1.082–14.945)	0.700	…	…
Arterial phase enhancement pattern	2.080 (0.197–22.003)	0.011	3.626 (0.505–26.036)	0.200
Dot−/band-like enhancement inside the tumor	0.308 (0.0566–1.676)	0.089	…	…
Visible vessel penetration	0.873 (0.087–8.743)	0.099	…	…
Visible hepatic artery penetration	6.835 (0.878–53.191)	0.001	2.249 (0.609–8.313)	0.224
Visible portal vein penetration	0.472 (0.081–2.763)	0.629	…	…
Visible hepatic vein penetration	3.730 (0.564–24.651)	0.111	…	…
Peripheral hepatic enhancement	0.302 (0.054–1.676)	0.615	…	…
Maximum diameter	0.991 (0.951–1.032)	0.001	1.013 (0.991–1.036)	0.240
Arterial maximum thickness	1.315 (1.019–1.696)	0.354	…	…
Arterial enhanced edge integrity	0.392 (0.075–2.057)	0.110	…	…
Arterial edge enhancement ratio (%)	0.874 (0.766–0.996)	0.010	0.995 (0.966–1.025)	0.760
Delayed phase enhancement ratio	1.960 (0.819–4.687)	0.812	…	…

… not included in multivariate analysis

However, in a multivariate logistic regression analysis, none of the above eight risk parameters were found to be independent risk factors for a diagnosis of MVI in ICCs (*P* > 0.05) (Table 5).

## Discussion

The results of our study indicate that six MR characteristics—four qualitative features (tumor morphology, intrahepatic duct dilatation, arterial phase enhancement pattern, and visible hepatic artery penetration sign) and two quantitative parameters (the maximum diameter of the tumor and the arterial phase edge enhancement ratio)—are associated with MVI of ICC. In addition, one tumor marker (CA19–9 level) and the pathological tumor grade are related to MVI.

Our study results show that a higher CA19–9 level (≥37 U/ml) is a risk factor that increase the probability of MVI in patients with ICC. This result is different from that reported by Zhou et al. [12]. This difference may be because the date range of the selected cases differs; in addition, as the number of cases in our study is small, some selection bias must be acknowledged. Therefore, large multicenter studies are needed. Recent studies have shown that the CA19–9 (≥500 U/ml) level was independently associated with poor overall survival in patients with ICC, and the CA19–9 level was used to guide the choice of treatment regimen [16–18]. Thus, there may be a significant correlation between MVI and prognosis; further follow-up and survival analyses are warranted.

Our study shows that tumor grade is also a risk factor associated with MVI. Grade G3 tumors accounted for 88.24% of MVI-positive ICC cases, indicating that a high proportion of MVI-positive tumors are poorly differentiated. Ali et al. found that poor tumor differentiation was associated with a significant decline in survival among patients with ICC [10]. Therefore, there may be a significant correlation between tumor MVI positivity and decreased survival in ICC patients, which should be explored further.

The results of our study also reveal that tumor morphology is a risk factor associated with MVI. Spherical tumors (including hemispherical and oval tumors) accounted for 44.59% of MVI-negative ICC cases but only 17.65% of MVI-positive ICC cases (Table 3), indicating that in MVI, spherical (including hemispherical and oval) tumors are more likely to be MVI-negative than MVI-positive.

Intrahepatic duct dilatation is a risk factor associated with MVI (Fig. 2h). This result is different from the findings presented by Zhou et al. [12], perhaps because the date range of the selected cases in the two groups is different. In addition, the number of cases in our study is small, and thus, some selection bias exists. Large sample, multicenter studies are needed in the future.

Another risk factor is the maximum diameter of the tumor, with the incidence of MVI increasing as tumor size increases. This finding is consistent with the conclusions of Zhou et al. and Spolverato et al. [12, 19]. Our research also shows that 61.76% of tumors in MVI-positive ICC patients and 35.14% of those in MVI-negative patients have a maximum diameter of > 5 cm—a significant difference (*P* = 0.010). Hence, in ICC, tumors with a maximum diameter of > 5 cm are more prone to exhibit MVI. Furthermore, Ali et al. showed that a tumor size > 5 cm was associated with a significant decrease in patient survival [10].

Our findings further suggest that the arterial phase enhancement pattern is a risk factor associated with MVI. Lesions showing overall enhancement in the arterial phase are more likely to be MVI-negative than MVI-positive (Fig. 5), while those showing partial enhancement (mixed signal) or no/mild enhancement (low signal) are more likely to be MVI-positive (Table 4, Figs. 2 and 4). The arterial edge enhancement ratio was also a risk factor for MVI (P = 0.010, Table 5), but the delayed phase enhancement ratio was not. ICCs with a lower arterial edge enhancement ratio are more likely to be MVI-positive (Figs. 2 and 4), while ICCs with higher arterial edge enhancement ratio are more likely to be MVI-negative (Figs. 3 and 5). In addition, the median of the arterial edge enhancement ratio of ICCs with MVI was 9.47%, while that of ICCs without MVI was 21.48% (Table 4). The edge of the ICC mass was enhanced in the arterial phase, and the enhancement range gradually filled toward the center in the delayed phase, indicating that the center of the tumor contains fibrous components (Figs. 2 and 3). Tumors had bright signal intensity in the central area in T2W-FS imaging and were not enhanced in any phases, indicating a necrotic area (fluid-like features) with fibrosis (Figs. 2 and 4). In other words, ICC lesions that have relatively lower arterial phase edge enhancement ratios have relatively more central fiber components (with or without necrotic area) and are more likely MVI-positive. Previous studies have confirmed that abundant desmoplastic stroma, one of the most characteristic histological findings of ICC, plays an important role in promoting enhanced malignant behavior and therapeutic resistance in patients with cholangiocarcinoma [20–22]. Therefore, we believe that ICCs with low marginal enhancement ratios in the arterial phase may contain more fibrous components and exhibit MVI, and the prognosis may be worse. Further studies, including investigations into the impact of MVI on prognosis and the correlation between MR signs and prognosis, are necessary to confirm this hypothesis.

Our results also indicate that visible hepatic artery penetration (the presence of a penetrating hepatic artery in the lesion) inside the tumor is a risk factor associated with MVI (Fig. 2i). This characteristic is similar to that observed in hepatocellular carcinoma (HCC), according to studies published by Xu X and Zhao H et al. [23, 24]. Intratumoral arteries were defined as the discontinuous and tortuous arteries in tumors, which were considered to be related to the invasiveness of HCC. Intratumoral arteries the strongest predictor for MVI of HCC observed by contrast-enhanced computed tomography [24]. The rate of intratumoral arteries in all HCC patients with MVI (81.0%) was significantly higher than that of those without MVI (25.8%) [24]. Currently, the mechanism of the intratumoral arteries is unclear. Segal et al. found that a 91-gene signature consisting of cell proliferation and matrix invasion genes was associated with venous invasion, especially MVI [25]. Therefore, intratumoral arteries reflect the invasiveness of HCC on the basis of the gene expression profile. Perhaps the same is true in ICC.

Recent studies have shown that the presence of MVI in ICC is a significant adverse prognostic factor, and patients without factors such as MVI do not need adjuvant chemotherapy after R0 resection [10, 11]. Therefore, preoperative predictions of MVI based on imaging are highly important. Unfortunately, there have been no recognized standards for preoperative MVI risk assessment in ICC patients to date.

For the preoperative prediction of MVI in ICC, our study achieved certain results. Thus, we believe that using radiomics [26] in combination with clinical biochemical indicators allows for risk to be predicted in these patients. Of course, further research is needed to identify independent risk factors. Unfortunately, in this work, we did not identify MR features that could be used as independent risk factors for the presence of MVI in ICC.

Our study has several limitations. First, this is a single-center retrospective study, the overall incidence of ICC is low, and the sample size was relatively small (especially the number of MVI-positive cases) for multivariate analysis. The positive rate of MVI in 108 patients with single ICC who were enrolled in this study was 31.48% (34/108), which may be lower than the actual positive rate. Because this study is a retrospective analysis, selection bias exists. Unenrolled cases, such as other pathological types (periductal infiltrating, intraductal growing and mixed-type ICCs), multiple lesions, lesions that can only undergo interventional or radiotherapy (some of which have lost the opportunity for surgical treatment), recurrence after surgery, etc., are more likely to develop MVI. In addition, we lacked a sufficient number of samples to establish a validation group to confirm the risk factors. We are preparing to conduct a multicenter study with a larger number of cases for further investigation. Second, six MRI scanners with two field strengths from three vendors were used in data collection. The variations between the six scanners may cause differences in MRI quantitative measurements. This effect is more important and should be considered in the future multicenter study; in particular, quantitative morphological measurements (such as diameter and thickness of lesions) can be more conveniently and readily calibrated by using a standard phantom with fixed dimensions among different scanners. Despite the uncalibrated morphological measurements in this study, we found potential value in their ability to predict MVI in patients with ICC. Third, considering that different MR models have a greater impact on ADC value measurement, our study did not involve ADC values.

Previous studies have shown that MVI is one of the factors associated with lower postoperative survival of patients with ICC [10]. Studies of Tsukamoto et al. have shown that the absence of MVI is also an independent predictor of ICC cure (they defined “cure” as a recurrence-free survival period of ≥5 years for patients who underwent R0 resection for primary ICC.), and MVI has been used as a reference factor for adjuvant chemotherapy after ICC surgery [11]. Therefore, preoperative prediction of MVI allows patients and physicians to know in advance if they will be using adjuvant chemotherapy and plan an overall treatment plan in advance. Therefore, preoperative prediction of MVI in patients with ICC has certain clinical significance. Our research focuses on the correlation between imaging features and MVI, in order to predict MVI before surgery and provide more information to the clinic. If it is accurately predicted, it may be possible to use anti-tumor vascular drugs to assist treatment for MVI-positive groups to improve survival.

## Conclusion

In summary, for the preoperative prediction of MVI in ICC, some qualitative and quantitative data obtained from preoperative MRI, and preoperative CA19–9 level were significant. In addition, postoperative pathological tumor grade is also associated with MVI. Our study analyzed multiple morphological features and a small amount of quantitative data obtained from preoperative MRI to find the correlation between MR imaging features and MVI. However, more research is needed to identify MR characteristics that can be used as independent risk factors.

## Data Availability

Yes, all data are available.

## Abbreviations

AFP: Alpha-fetoprotein
CA19–9: Cancer antigen-19-9
CEA: Carcinoembryonic antigen
DWI: Diffusion-weighted image
Gd-DTPA: Gadopentate dimeglumine (Bayer HealthCare, Berlin, Germany)
Ga-EOB-DTPA: Gadoxetic acid (Primovist; Bayer Healthcare, Leverkusen, Germany)
HCC: Hepatocellular carcinoma
ICC: Intrahepatic cholangiocarcinoma
MRI: Magnetic resonance imaging
MVI: Microvascular invasion
RFA: Radiofrequency ablation
T1WI: T1-weighted image
T1W-FS: T1-weighted fat-suppressed
T2WI: T2-weighted image
T2W-FS: T2-weighted fat-suppressed
TACE: transcatheter arterial chemoembolization
